# Delivery of improved oncolytic adenoviruses by mesenchymal stromal cells for elimination of tumorigenic pancreatic cancer cells

**DOI:** 10.18632/oncotarget.7031

**Published:** 2016-01-27

**Authors:** Adam Kaczorowski, Katharina Hammer, Li Liu, Sabine Villhauer, Clifford Nwaeburu, Pei Fan, Zhefu Zhao, Jury Gladkich, Wolfgang Groß, Dirk M. Nettelbeck, Ingrid Herr

**Affiliations:** ^1^ Surgical Research Section, Molecular OncoSurgery, Department of General and Transplantation Surgery, University of Heidelberg and German Cancer Research Center (DKFZ), Heidelberg, Germany; ^2^ Oncolytic Adenovirus Group, German Cancer Research Center (DKFZ), Heidelberg, Germany

**Keywords:** TRAIL, virus therapy

## Abstract

Pancreatic ductal adenocarcinoma (PDA) is one of the most aggressive malignancies and has poor therapeutic options. We evaluated improved oncolytic adenoviruses (OAds), in which the adenoviral gene E1B19K was deleted or a TRAIL transgene was inserted. Bone marrow mesenchymal stromal cells (MSCs) served as carriers for protected and tumor-specific virus transfers. The infection competence, tumor migration, and oncolysis were measured in cancer stem cell (CSC) models of primary and established tumor cells and in tumor xenografts. All OAds infected and lysed CSCs and prevented colony formation. MSCs migrated into PDA spheroids without impaired homing capacity. Xenotransplantation of non-infected PDA cells mixed with infected tumor cells strongly reduced the tumor volume and the expression of the proliferation marker Ki67 along with a necrotic morphology. Adenoviral capsid protein was detected in tumor xenograft tissue after intravenous injection of infected MSCs, but not in normal tissue, implying tumor-specific migration. Likewise, direct *in vivo* treatment correlated with a strongly reduced tumor volume, lower expression of Ki67 and CD24, and enhanced activity of caspase 3. These data demonstrate that the improved OAds induced efficient oncolysis with the OAd-TRAIL as most promising candidate for future clinical application.

## INTRODUCTION

Pancreatic ductal adenocarcinoma (PDA) is one of the most aggressive malignancies [1]. The poor prognosis is attributed to cancer stem cells (CSCs), which survive conventional cytotoxic therapy due to defense and survival mechanisms [2].

Viral treatment of cancer is an old concept that has been revisited in recent years using genetically modified viruses, including oncolytic adenoviruses (OAds). OAds are in clinical development for the treatment of various cancers because the replication of the viruses can be restricted to malignant cells [3–5]. OAds replicate in tumor cells; at the end of the viral replication cycle, the cell is lysed, and new infectious virus particles are released, which then infect and lyse neighboring tumor cells. The therapeutic potential involves the activation of endogenous tumor immune responses [6]. A major advantage of OAds is their excellent safety profile, which has been demonstrated in multiple studies of patients with ovarian cancer, melanoma, soft tissue or primary bone sarcoma and other neoplasms [3-5, 7-12]. In engineered OAds, the virus capsid is modified to improve binding to tumor cells [13]. Virus carriers have been studied to shield viruses against inactivation by neutralizing antibodies present in many patients from earlier adenovirus infections [14]. Mesenchymal stromal cells (MSCs) have been used as carriers due to their reported tumor-specific homing capacity, which enables the protected transfer and release of the viruses on site [14–17]. Castleton and colleagues [18] provided evidence that MSCs can be efficiently used as carriers for systemic delivery of oncolytic measles virus to treat acute lymphoblastic leukemia, even in the presence of high-titer neutralizing antibodies. Of particular interest is a small pilot study of OAd-delivery by MSCs in four children with metastatic neuroblastoma refractory to front-line therapies [19]. The patients received several doses of autologous MSCs carrying ICOVIR-5, an AdΔ24RGD-derivative OAd with tumor specificity that was designed to increase replication potency in tumor cells [20]. The tolerance to the treatment was excellent, and a complete clinical response was documented in one case. The child remained in complete remission 3 years after this therapy. Current efforts concentrate on increasing the efficacy of OAds, e.g., by inserting therapeutic genes [21] to support tumor lysis by inducing apoptosis or an anti-tumor immune response [6].

Recently, we genetically engineered adenoviruses for improved delivery by MSCs with the aim of enhancing oncolysis and improving virus production in PDA cells [22]. In this prior study, the capsid of adenovirus serotype 5, from which regular OAds are derived, was modified, resulting in consistently strong infection of PDA cells and MSCs [22]. The adenovirus was modified by inserting the chimeric Ad5/3 fiber, in which the knob domain with the HAdV-5 fiber has been replaced by the corresponding domain of the HAdV-3 fiber, binding to the widely expressed desmoglein-2 [22]. Thus, cell entry is independent of coxsackievirus-adenovirus receptor (CAR), which is poorly expressed on the cell surface of PDA cells and MSCs [22]. In addition, the replication and release of OAds in infected cells was improved by deleting the pro-apoptotic early adenoviral gene E1B19K or expression of the full-length human TRAIL gene from a viral transcription unit. The deletion of E1B19K has been reported to increase viral release and spread and, consequently, the therapeutic efficacy in experimental PDA models [23]. TRAIL was inserted because this tumor-specific death ligand induces paracrine apoptosis in neighboring tumor cells by eliminating non-infected tumor cells and autocrine effects presumably responsible for improved viral cell lysis and release in infected MSCs and PDA cells. Also, TRAIL exhibited low side effects in phase I/II clinical studies [24]. The resulting viruses Ad5/3-Δ19K-.Luc and Ad5/3-TRAIL demonstrated strongly increased total viral particle production and release from MSCs compared to the parental virus Ad5/3-Luc [22].

In the present study, we investigated the therapeutic efficacy of the improved OAds in established and primary CSC marker-enriched PDA cell lines after administration alone or via MSC carriers by *in vitro* invasion assays and xenotransplantation studies. The Ad5/3-TRAIL construct enabled effective tumor invasion by OAd-MSCs in spheroids and xenografts and significant *in vivo* elimination of tumorigenic cells.

## RESULTS

### Oncolytic adenoviruses infect primary pancreatic CSCs

**Figure 1:**
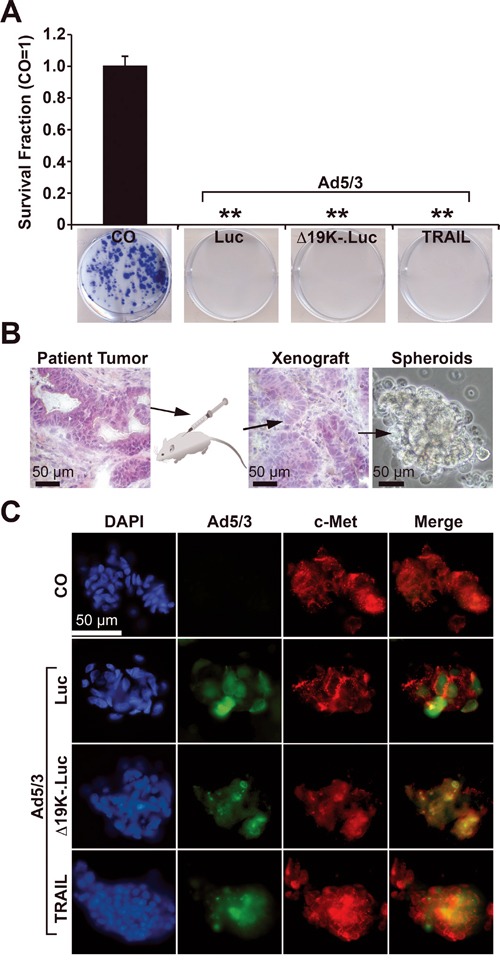
Infection of pancreatic CSCs by OAds **A.** MIA-PaCa2 cells were infected at a titer of 200 TCID_50_ with Ad5/3-Luc (Luc), Ad5/3-Δ19K-.Luc (Δ19K-) or Ad5/3-TRAIL (TRAIL), or were left uninfected (CO). Twenty-four hours later, the cells were re-plated at a low density (400 cells/well) in 6-well plates. After two weeks, colonies containing more than 50 cells were counted using a dissecting microscope. The number of surviving colonies in the control was set to 1, and the survival fraction is presented. **B.** Tumor cells were freshly isolated from a mouse xenograft derived from the primary, low-passage, CSC-enriched human PDA xenograft cell line T30. The morphologies of the patient tumor and its mouse xenograft in passage 9 were detected by H&E staining and immunohistochemistry. The cells were cultured in an anchorage-independent manner as spheroids *in vitro*, which promotes the growth of CSCs (photograph on the right). **C.** Primary tumor spheroids were uninfected (CO) or infected at a titer of 200 TCID_50_ with Ad5/3-Luc (Luc), Ad5/3-Δ19K-.Luc (Δ19K-) or Ad5/3-TRAIL (TRAIL). Spheroids were fixed and centrifuged on slides 42 h after infection. Adenoviral capsid protein (green), expression of the CSC marker c-Met (red), or both together (merge, yellow) were detected using specific antibodies and double immunofluorescence staining. The cell nuclei were counterstained with DAPI (blue). Representative images at 400× magnification are shown.

To study the influence of OAd constructs on the potential for self-renewal, MIA-PaCa2 cells were infected, and live cells were re-seeded at clonogenic density 24 h later. After 2 weeks, the non-infected cells had formed colonies, but no colonies were detected in cells infected with the regular OAd construct Ad5/3-Luc or with the improved OAd constructs Ad5/3-Δ19K-.Luc or Ad5/3-TRAIL (Figure 1A, compare to Table 1), suggesting that the cells were already completely lysed by the parental virus. To evaluate the invasion potential of the OAd constructs in three-dimensional (3D), primary CSC spheroids, tumor cells were isolated from patient-derived PDA tissue by serial transplantation in mice and subsequent spheroidal culture (Figure 1B). These primary tumor spheroids are highly enriched in CSC markers [25]. The spheroids were infected, and 24 h later, the presence of adenoviral capsid protein, which reflects the amount and location of viruses, was detected by staining with a specific antibody. In addition, the expression of the CSC marker c-Met was detected by double immunofluorescence staining. Green-fluorescent-labeled adenoviral capsid protein was detected only in infected cells and not non-infected control cells (Figure 1C). By contrast, the red-fluorescent c-Met protein was present in infected and non-infected cells, demonstrating the CSC character of the spheroidal cell model. Double-stained, adenoviral capsid and c-Met-positive, yellow fluorescent cells were present in high amounts. These results indicate that all tested OAds successfully prevent colony formation and spread in 3D primary CSC spheroids, and no advantage of the improved OAds was detectable under these *in vitro* conditions.

**Table 1:** Properties of the used OAd constructs

Virus name	Abbreviation	Properties	Reference
Ad5/3	-	-	present work
Ad5/3-Luc	Luc	Luciferase gene fused via IRES to the fiber gene.	[23]
Ad5/3-Δ19K-.Luc	Luc/19K-	Luciferase gene fused via IRES to the fiber gene, deletion of E1B19K.	[23]
Ad5/3-TRAIL	TRAIL	Human TRAIL gene fused via IRES to the fiber gene.	[23]

All viruses have a 24 amino acid deletion in E1A for tumor-selectivity, an E3-deletion, an Ad5/3 chimeric fiber for enhanced cell entry and a GFP gene upstream of RITR, expressed from the major late promoter.

### OAd-infected MSC carriers invade tumor spheroids

For protected and tumor-specific transfer, we used as carriers MSCs that were isolated from the bone marrow of different healthy donors, expanded, and selected. In passage 3, the progenitor cell character of the MSCs was confirmed according to the minimal criteria for defining multipotent mesenchymal stromal cells of the International Society for Cellular Therapy (ISCT). Thus, the MSC were plastic-adherent in standard culture conditions, they expressed a characteristic surface marker pattern of CD34^−^, CD45^−^, CD166^−^, CD44^+^, CD90^+^ and CD105^+^ and they differentiated to osteoblasts, adipocytes and chondroblasts *in vitro* (SFig. 1). Therefore, the MSCs were used for further experiments between passages 4 and 8.

**Figure 2:**
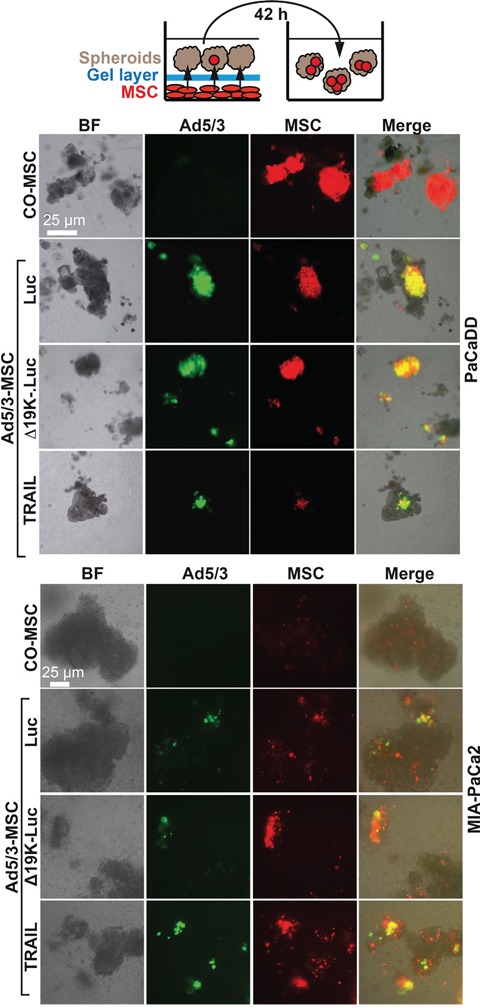
OAd-infected MSC carriers invade tumor spheroids MSCs growing as a monolayer in 24-well plates were stained with the red fluorescent dye PKH26; 2 μM dye solution (provided by the manufacturer) was added per well. MSCs were infected with Ad5/3-Luc (Luc), Ad5/3-Δ19K-.Luc (Δ19K-) or Ad5/3-TRAIL (TRAIL) at a titer of 2000 TCID_50_ 24 h later. Two hours after viral infection, the MSCs were covered with a 1:1:1 mixture of Matrigel, collagen and methylcellulose in DMEM medium supplemented with 2% FCS. Immediately afterward, tumor spheroids prepared from the primary, PDA cell line PaCaDD in passage 10 or from the established PDA cell line MIAPaCa-2 were seeded on top of the gel layer. The cells were co-incubated for 16 h to allow invasion of the MSCs into the spheroids through the gel layer. The spheroids were then transferred into new culture plates using a pipette and cultivated until 42 h after infection. Invasion was evaluated by detection of adenoviral capsid protein (green) by staining with a specific antibody and double immunofluorescence microscopy. Bright field (left), fluorescence (right: Adenoviral capsid protein: green; MSCs: red; Merge: yellow). Representative images at 400× magnification are shown.

To evaluate the attraction of OAd-infected MSCs to tumor spheroids *in vitro*, MSCs attached to the bottom of 24-well plates were marked with the red fluorescent dye PKH26. Twenty-four hours later, the MSCs were infected or left uninfected with parental Ad5/3-Luc or the improved Ad5/3-Δ19K-.Luc or Ad5/3-TRAIL viruses, and the cells were covered with a gel layer on which spheroids from PaCaDD183 or MIA-PaCa2 cells were seeded and co-incubated for 16 h (Figure 2). Forty-two hours after infection, that is, 6 h before viral lysis [22], the spheroids were separated, and the integration of red MSCs was analyzed by immunofluorescence microscopy. Red fluorescent MSCs were detected in all co-incubated spheroids, suggesting that infected- and non-infected MSCs were attracted. However, double-fluorescent, yellow, virus-expressing MSCs were only detected in spheroids co-incubated with infected MSCs, as expected. This result suggests the successful migration of MSCs in 3D spheroidal structures.

### OAd *in vitro* infection prevents tumorigenicity

**Figure 3:**
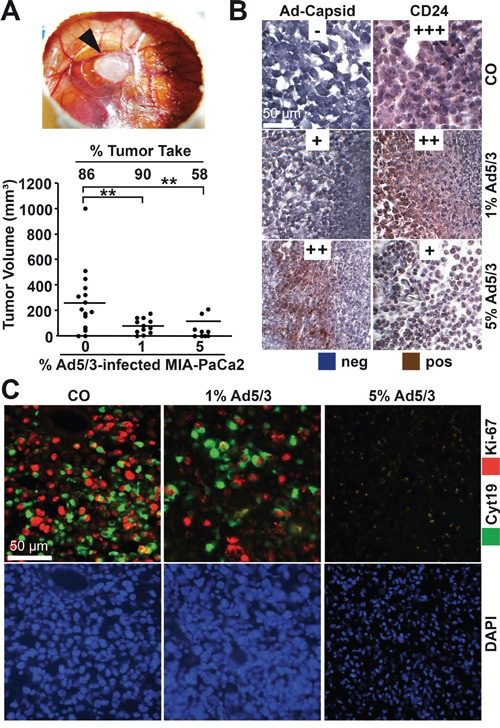
OAd-infected PDA cells exhibit reduced tumorigenicity **A.** MIA-PaCa2 cells were uninfected or infected with Ad5/3. Two hours later, 5 × 10^5^ uninfected cells (CO) or ratios of 99:1 (1) or 95:5 (5) 5 × 10^5^ uninfected:infected MIA-PaCa2 cells were transplanted to the CAM of fertilized chick eggs at day 9 of development (15 eggs per group). The results for xenografted eggs with live chick embryos were evaluated. The black arrow marks tumor xenograft formed by uninfected MIA-PaCa-2 cells at day 18 of development. The engraftment efficiency expressed as the % tumor take of transplanted tumors and the tumor volumes were determined, and the single data points and the means of each group are shown (**P < 0.01). **B.** The presence of adenoviral capsid protein (Ad-Capsid) and of the CSC marker CD24 was evaluated by staining with specific antibodies and immunohistochemistry. To evaluate expression levels, a semi-quantitative scoring system was used based on the determination of the percentage of positive cells by visual inspection: high (+++), medium (++), low (+) and absent (−). **C.** Double immunofluorescence staining of xenograft tissue with a human-specific antibody against cytokeratin 19 (Cyt19, green) and the proliferation marker Ki67 (red). Cell nuclei were counterstained with DAPI (blue). Representative images at 400× magnification are shown.

**Figure 4:**
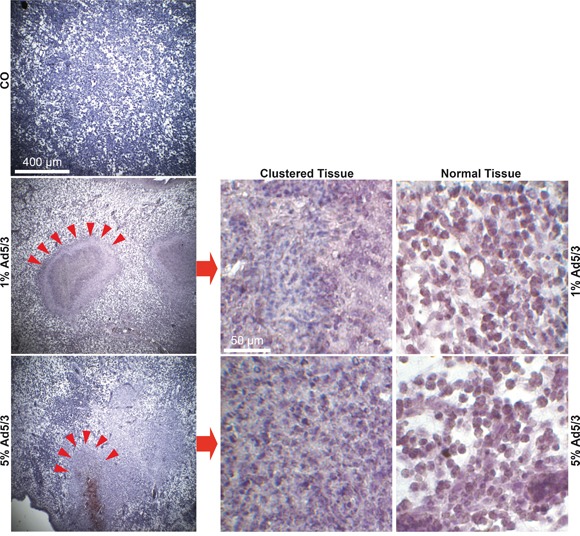
OAd treatment leads to clustered cell regions MIA-PaCa2 cells were uninfected or infected with Ad5/3. Two hours later, 5×10^5^ uninfected cells (CO) or ratios of 99:1 (1) or 95:5 (5) 5×10^5^ uninfected:infected MIA-PaCa2 cells were transplanted to the CAM of fertilized chick eggs at day 9 of development (15 eggs per group). Tissue sections from the resected xenograft tumors were prepared and stained with a human-specific antibody against cytokeratin 19 (Cyt19). Representative images are shown at 40× magnification, and compact, cluster-like structures are marked with red arrows. Details of cluster-like structures (Clustered Tissue) and normal tissue structures (Normal Tissue) are shown at 400× magnification.

To assess the influence of OAd infection on tumorigenicity, we first used a control Ad5/3 construct to establish the detection of viral spread in xenograft tumors. MIA-PaCa2 cells were xenotransplanted in fertilized chick eggs. This method is an *in vivo* replacement method for mouse studies. Chick embryos are naturally immunodeficient because full immunocompetence in birds develops only after hatching at day 21 of development [26]. Xenografted tumors are well supplied by blood vessels from the CAM, and chick fibroblasts participate in the formation of the tumor stroma. Most importantly, the CAM of the chick embryo is not innervated; therefore, unlike mice, the embryo does not feel pain during transplantation and tumor growth. Favorably, this system has no administrative barriers, it is inexpensive and well suited for short-term *in vivo* xenograft studies. MIA-PaCa2 cells were transplanted in the CAM of fertilized chick eggs, either as uninfected cells or in cell mixtures containing 1% or 5% infected cells. Tumor xenografts developed rapidly from uninfected control cells 9 days after transplantation (Figure 3A). The embryos were humanely euthanized, followed by tumor resection and calculation of the percentage of engrafted xenografts and tumor volumes. The tumor take rate was 86% in the control group and 90% for tumor cells containing 1% infected MIA-PaCa2 cells but decreased to 58% in the presence of 5% infected cells. The adenoviral capsid protein was present in tumor samples of infected xenografts, as shown by immunohistochemistry (Figure 3B). Simultaneously, the progression marker CD24 was reduced in infected tumors compared to uninfected controls, demonstrating the reduction of CD24-expressing tumor cells by the OAd virus. In addition, proliferation was strongly reduced in 1% infected cells and nearly undetectable in 5% infected cells, as concluded from double immunofluorescence microscopy with the human-specific markers Ki67 and cytokeratin 19 (Figure 3C). Moreover, DAPI staining of the cell nuclei revealed strongly shriveled nuclei in tumors containing 5% infected cells, indicating complete elimination of tumorigenic cells. Interestingly, the morphology of xenograft tissue from infected cell mixtures differed from that of uninfected control xenografts and contained densely packed, small cells located in cluster-like structures (Figure 4), suggesting that these are the regions in which the virus spread and lysed the cells.

### OAd-TRAIL is most therapeutically significant virus *in vivo*

**Figure 5:**
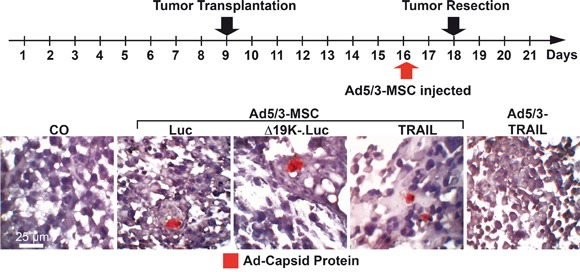
OAd-infected MSCs invade tumors *in vivo* At developmental day 9, 5×10^5^ MIA-PaCa2 cells were transplanted to the CAM of chick embryos (18 eggs per group). At day 16, the CAM was injected with 100 μL of cell culture medium (CO), 1×10^8^ TCID_50_ Ad5/3-TRAIL in 100 μL of cell culture medium, or 5×10^4^ MSCs in 100 μL of cell culture medium 2 h after infection with 2000 TCID_50_ of Ad5/3-Luc (Luc), Ad5/3-Δ19K-.Luc (Δ19K-) or Ad5/3-TRAIL (TRAIL). The tumor xenografts were resected at day 18. Tissue sections were prepared and paraffin-embedded, and the expression of adenoviral capsid protein (red) was detected by immunohistochemistry. Representative images at 400× magnification are shown.

**Figure 6:**
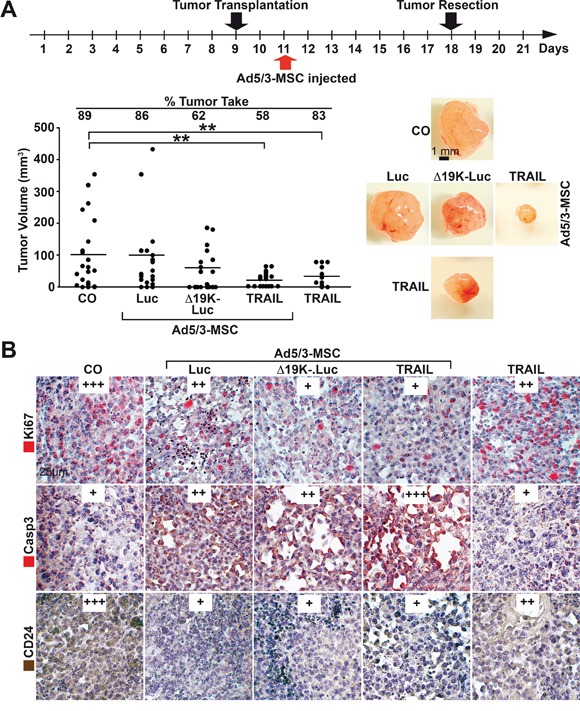
MSC-delivered OAds reduce tumor growth *in vivo* **A.** Experimental procedure: MIA-PaCa2 cells (5×10^5^) were transplanted to the CAM of chick embryos at developmental day 9 (20 eggs per group), followed by therapeutic injection at day 11, as described in Figure 5, and tumor resection at day 18. The tumor take rate and the volume of the resected xenografts were determined, and the single data points and the means of each group are shown (**P<0.01) along with representative images of tumor xenografts of each group. **B.** Sections were prepared from the xenograft tumors and paraffin-embedded, and the expression of Ki67, cleaved fragment of Caspase 3 (Casp3) and CD24 were examined by immunohistochemistry. Representative images are shown at 400× magnification. The expression levels were evaluated by a semi-quantitative scoring system as described in Figure 4B.

To compare the invasion, spread and lysis mediated by the systemic delivery of infected MSCs, Mia-PaCa2 cells were transplanted to the CAM of fertilized chick eggs. Seven days later, the xenografted eggs were left untreated, or the CAM vessels were injected with virus-infected MSCs or the Ad5/3-TRAIL virus without carrier (Figure 5). Two days later, the tumors were resected to examine the virus distribution in the xenograft tissue. Tissue sections were prepared and stained with a specific antibody to detect adenoviral capsid protein. Positive signals were detected in xenograft tissue from eggs injected with virus-infected MSCs but not in xenografts from eggs injected with Ad5/3-TRAIL virus alone or in control xenografts. By contrast, adenoviral capsid protein was not detectable in the xenografts of non-injected eggs, as expected, or in xenografts from eggs injected with the Ad5/3-TRAIL-GFP virus alone without MSC carriers. This result suggests that the delivery of virus with MSCs was more efficient, and most likely several particles were present in the same tumor region and thereby enabled the detection by immunohistochemistry. We cannot rule out that virus particles were also present in tumor tissue after transfer of the virus alone, but the isolated particles may have been too tiny and thus under the detection limit of immunohistochemistry. Because significant oncolysis occurred after delivery of virus without MSCs, the virus must have been replicated and spread with a time delay compared to MSC-delivered virus (Figure 6A). As a control for tumor-specific virus distribution, we stained normal chick embryonal tissue with an antibody for adenoviral capsid protein, but we were not able to detect virus particles in liver, heart and lung after virus delivery with or without MSCs (data not shown), suggesting that the delivery was tumor-specific. Also, we were not able to prove the presence of MSCs in tumor tissue, due to a lack of a specific single marker that would distinguish MSCs from tumor fibroblasts. However, the fact that adenoviral capsid protein was detectable in tumor tissue after transfer by MSCs only strongly supports the conclusion that virus-infected MSCs migrated into the tumors.

To assess tumor inhibition, MIA-PaCa2 cells were transplanted, but the therapy was injected later than before, at day 11, in CAM blood vessels to allow virus spread and lysis until the tumors were resected at day 18 (Figure 6A). This strategy resulted in a strong reduction of the engraftment rate from 89% in the control group to 62% and 58% in the groups that received MSC carriers with Ad5/3-Δ19K-.Luc or Ad5/3-TRAIL, respectively. However, MSC carriers with the parental Ad5/3-Luc virus or application of the Ad5/3-TRAIL virus alone reduced the tumor take rate only minimally. The Ad5/3-TRAIL virus effectively reduced the tumor volume whether transferred with or without MSCs, suggesting that the virus alone also homed to the tumor xenografts, but viral spread and lysis occurred with a time delay. Toxicity to the chick embryos was not observed, as expected due to the human-specific nature of the OAds. Immunohistochemistry of tumor sections demonstrated that all groups treated with OAds had reduced viability, as determined by Ki67 staining, and the strongest inhibition was mediated by the two improved OAds Ad5/3-Δ19K-.Luc and Ad5/3-TRAIL transferred by MSCs (Figure 6B). Similarly, the Ad5/3-TRAIL virus transferred by MSCs was superior at inducing apoptosis, as shown by staining of the active fragment of caspase-3. Additionally, all virus-treated groups had reduced expression of the progression marker CD24, with strongest and comparable effects in xenografts of eggs injected with OAds in MSC carriers. Taken together, our findings reveal that MSC delivery of OAds is superior to the delivery of OAds alone in tumor therapy. Among the two improved OAds tested, the Ad5/3-TRAIL virus was superior, most likely due to the paracrine induction of apoptosis in parallel to virus-induced tumor cell lysis.

## DISCUSSION

In the present preclinical study, we investigated the therapeutic suitability of improved OAds with the aim of targeting highly therapy-resistant pancreatic stem-like tumor cells. The genetic engineering of OAds specific for PDA and MSC carriers and their infection and lysis efficiency were recently described by us [22]. The present study focused on the elimination of tumorigenicity, tumor growth and progression markers *in vivo* after administration of the improved OAds alone or via MSC carriers. We examined the Ad5/3-Δ19K-.Luc construct, which features a deletion of the viral gene E1B19K to permit increased viral release and spread [23], and the Ad5/3-TRAIL construct to exploit viral lysis by autocrine TRAIL activity and complement it with inducing paracrine apoptosis in neighboring, non-infected tumor cells.

Whereas our former publication demonstrated the successful infection of spheroidal growing established PDA cell lines [22], the present study showed the successful OAd infection of primary CSC spheroids from PDA patient tumors. The OAd constructs completely prevented the self-renewal potential, which is further underlined by our observation that no colonies were formed after infection of the established PDA cell line MIA-PaCa2. No obvious differences among the three viruses used were detectable under these *in vitro* conditions. The present data extend our prior findings demonstrating the successful infection of spheroidal cultures formed by MIA-PaCa2 cells [22]. Moreover, these data confirm recent reports in which an effective adenoviral lysis of primary CSCs derived from breast cancer was observed both *in vitro* and in orthotopic tumors in mice [27]. These results support the suitability of OAds for future therapeutic targeting of CSCs, in agreement with a recent review article suggesting oncolytic viral therapy for the targeting of CSCs [28]. Because CSCs are suggested to be the root of cancer growth, spread and recurrence [29], only the elimination of these tumor progenitors may provide a cure for patients with PDA.

Recent clinical studies have demonstrated inefficient delivery of oncolytic viruses by systemic application. This inefficiency is due to inactivation of viruses by the immune system and clearance from the blood by liver macrophages [30]. To circumvent this problem, we transferred the engineered OAds via MSC carriers because these progenitor cells have tumor tropism [31], sustain the replication of OAds [22], especially of the improved OAds used here [22], and were recently shown to release infectious particles in orthotopic lung and breast tumors [32]. The positive results of our invasion assays, in which infected MSC virus carriers successfully integrated through a gel layer into 3D spheroidal cultures of primary and established PDA cell lines, demonstrate that MSCs retain their tumor tropism and migration activity to overcome connective tissue-like structures despite OAd infection. This finding is particularly important because the extremely dense fibrotic matrix of PDA prevents even small therapeutic molecules from entering and perfusing [33] and is likely responsible for the marked resistance to chemo- and radiotherapy [34]. Another *in vitro* study has highlighted the advantage of adenoviral vector delivery by MSCs and observed increased delivery to breast and ovarian tumor xenografts in mice, with negligible levels of systemic toxicity compared with administration of adenovirus alone [35]. In preparation for a phase I clinical trial in ovarian cancer, significantly prolonged survival was observed in ovarian cancer-bearing mice treated with measles virus-infected MSCs from the adipose tissue of healthy donors and ovarian cancer patients but not those treated with the virus alone [36].

To detect differences between our OAd constructs, we examined the viral efficiency in a chick egg tumor xenograft model. Compared to mice, the egg system has several advantages, when used for short-time transplantation of 10 days. The tumor cells are transplanted to the chorioallantoic membrane (CAM), which is a well-vascularized extra-embryonic tissue located underneath the eggshell. According to our experience, the chick blood vessels quickly supply the tumor cells with blood. The chick embryo is naturally immunodeficient and the CAM readily supports the engraftment of tumor cells. Up to day 18 of chick development an animal application is not required, and the costs for the chick system are much lower compared to the mouse system. The experiments can be performed in a normal laboratory by the use of inexpensive egg incubators. Tumor xenografts growing on eggs have a pronounced tumor stroma, which consists of human tumor cells, chick fibroblasts, chick vessels and other host cells and thus provide the advantage of a tumor stroma, similar to the mouse xenotransplantation system. In a first approach, we evaluated the effect of a regular OAd virus infected *in vitro*, followed by xenotransplantation of a small amount of infected cells mixed with uninfected cells. This strategy was chosen because the uninfected cells can form tumors until the virus from the infected cells starts to replicate, lyse and spread to neighboring tumor cells embedded in the protective tissue structure. Indeed, we observed strongly decreased tumor engraftment and tumor volume as well as morphological changes in the virus-bearing tumor tissues, characterized by clusters of dense tissue. These dense clusters are likely the result of a cytopathic virus effect, which has been reported in response to adenovirus infection [37]. Consistent with these effects, the proliferation and expression of the CSC marker CD24 was decreased, and hardly any CD24-positive or proliferating cells were found in the dense clusters. We proceeded to transfer the OAd viruses, either alone or in MSCs, by systemic injection into blood vessels a few days after the tumor xenografts were transplanted. Viral capsid protein was detected in tumor tissue two days after injection, suggesting successful transfer by MSCs. By contrast, adenoviral capsid protein could not be detected in tumor tissue after the transfer of virus alone, or in embryonic tissue, suggesting that the virus transfer without MSC was less efficient and the delivery by MSC was specific for tumor tissue. Similar observations were made in a previous study demonstrating homing of virus-infected MSCs to different tumor types, including tumors with poor accessibility, such as glioma [15, 38]. Our results indicate that both of the improved OAds strongly reduced the expression of markers for stemness (CD24) and progression (Ki67) and enhanced the presence of the apoptosis marker “cleaved fragment of active caspase-3” after delivery by MSCs *in vivo*. Although our recent *in vitro* data suggested that the improved 19K-depleted OAd was superior regarding virus replication and release [22], the present *in vivo* data show that the OAd-TRAIL exhibited a superior effect after MSC delivery compared to delivery of OAd-TRAIL alone or compared to MSCs infected with the 19K-depleted OAd or with regular OAds. This conclusion was based on the strong and significant potential to reduce the tumor take rate and tumor volume and demonstrates the prominent bystander effect mediated by OAd-TRAIL. The enhanced efficiency of OAds expressing TRAIL is supported by other studies in which the viruses were not transferred by MSCs [39, 40]. Another advantage of TRAIL transfer by OAds is the known potential of TRAIL to induce apoptosis in senescent and malignant cells while showing no cytotoxicity in untransformed cells [41]. This finding is important because prior studies have suggested a tumorigenic potential of MSCs [42, 43]. However, this can be prevented by our strategy: MSCs are lysed by OADs after tumor-specific transfer. To address the toxicity and virus distribution in individuals with advanced PDA, an initial phase I dose escalation study using intratumoral injections of the OAd VCN-01 with or without gemcitabine has just started in Spain and is recruiting patients (NCT02045589). Similar to our OAd-TRAIL, the VCN-01 construct combines selective tumor replication with an improved tropism by insertion of the integrin-binding RGD peptide into the fiber. Instead of TRAIL, it expresses the hyaluronidase gene to degrade the dense extracellular tumor matrix and belongs to human adenovirus serotype 5 [44].

Taken together, our results demonstrate that delivery of the new OAd Ad5/3-TRAIL construct by MSC carriers induces a strong anti-tumor response in our experimental system. This therapeutic approach was even effective in tumorigenic cells with CSC characteristics, which do not respond to conventional chemotherapy. An important advantage of OAd Ad5/3-TRAIL is the selective replication in MSCs and PDA cells and the expression of TRAIL to induce tumor-specific apoptosis in non-infected cells. Oncolysis and the induction of apoptosis may induce an advanced inflammatory response and thus “open” the tumor microenvironment for conventional cytotoxic therapy or innate immune attacks. The clinical feasibility of such systemic MSC-OAd therapy warrants clinical investigation and may be understood as an initial aid to disrupt the dense tumor stroma for tumor cell eradication by innate immune responses and/or cytotoxic tumor therapy.

## MATERIALS AND METHODS

### Cell lines

The established human PDA cell line MIA-PaCa2 was obtained from American Type Culture Collection (ATCC, LGC Standards, Molsheim Cedex, France) and cultured in DMEM (18 mmol/l glucose) supplemented with 10% FCS and 5% HEPES. Human primary bone marrow-derived MSCs and the primary PDA cell line PaCaDD183 were isolated and cultured as previously described [45, 46]. The primary PDA xenograft line T30 was isolated from surgical non-diagnostic specimens by serial transplantation in mice and subsequent anchorage-independent *in vitro* culture, resulting in the selection of a CSC population as previously described [26]. All cells were grown in a humidified incubator at 37°C and 5% CO_2_ and authenticated throughout culture by their typical morphology. To maintain the authenticity of the cell lines, frozen stocks were prepared initially, and every three months a new frozen stock was used for experiments. MIA-PaCa2 cells were recently authenticated by a commercial service (Multiplexion, Heidelberg, Germany). Mycoplasm-negative cultures were ensured by performing monthly mycoplasm tests.

### Isolation of human MSCs from bone marrow and culture

Human bone marrow was harvested from the iliac crest of 2 young healthy donors between 20 and 30 years old. 40 mL bone marrow aspirate were collected in a syringe containing 10,000 IU heparin to prevent coagulation. The mononuclear cell fraction was isolated by Biocoll density-gradient centrifugation (d = 1.077 g/cm^3^; Biochrom, Berlin, Germany). Cells were plated on fibronectin (10 ng/mL, Sigma, Taufkirchen, Germany)-coated cell culture flasks and selected by plastic adherence upon culture in MSC-NH expansion medium (Miltenyi Biotec, Bergisch Gladbach, Germany).

### Flow cytometry (FACS) analysis of MSC marker expression

FACS analysis of marker expression was performed as described [45].

### Recombinant oncolytic adenoviruses

Improved OAds were constructed as described [22]. The new OAd construct Ad-5/3 was cloned in the present study by inserting a GFP gene (from plasmid pEGFP-N1, Clontech, Saint-Germain-en-Laye, France) into the plasmid pSΔ24 [47], followed by homologous recombination with plasmids containing the Ad5/3ΔE3 genome. In all virus strains, the capsid contains a fiber 5/3 chimera with the shaft of serotype 5 and the knob domain of serotype 3. All constructs contain a GFP gene in the E4 locus. Other properties of the virus constructs are described in Table 1.

### Colony-forming assay

Twenty-four hours after OAd infection of MIA-PaCa2 cells with 200 TCID_50_, 400 cells/well in 6-well tissue culture plates were re-seeded in complete medium and incubated for 2 weeks without changing the medium. The cells were then fixed with 3.7% PFA for 10 min and 70% ethanol for 10 min. After washing with distilled water, the cells were stained with 0.05% Coomassie Blue for 5 min. Then, the cells were washed with distilled water and dried overnight. Colonies with more than 50 cells were counted under a dissecting microscope.

### Establishment and infection of primary tumor spheroids

Spheroids obtained from the primary xenograft line T30 were cultured in NeuroCult NS-A basal serum-free medium (human) (StemCell Technologies, Vancouver, Canada) supplemented with 2 μg/ml heparin (StemCell Technologies, Vancouver, Canada), 20 ng/ml hEGF (R&D Systems, Wiesbaden-Nordenstadt, Germany), 10 ng/ml hFGF-b (PeproTech, Hamburg, Germany) and 10% NeuroCult NS-A Proliferation Supplements (StemCell Technologies, Vancouver, Canada) and were used for experiments within 7 d after isolation. The cells were resuspended in DMEM medium containing 2% FCS for infection and incubated with 200 TCID_50_ (50% tissue culture infective dose) for 2 h before use in experiments.

### Invasion of MSC-delivered OAds into spheroidal tumor cells

PaCaDD or MIA-PaCa2 cells were seeded in DMEM cell culture medium containing 2% heat-inactivated FCS and 0.25% methylcellulose (PureCol, Biomaterials, Fremont, USA) in a 96-well suspension culture plate (Greiner Bio-One, Frickenhausen, Germany) with a density of 10,000 cells/well for spheroid formation. The cells were used for co-incubation experiments with infected MSCs 72 h later. MSCs were stained with the PKH26 red fluorescent cell linker kit for general cell membrane labeling (Sigma-Aldrich, Munich, Germany), and 5 × 10^4^ MSCs/well were seeded in a 24-well plate in DMEM cell culture medium supplemented with 2% FCS. The MSCs were infected 24 h later with a virus titer of 2000 TCID_50_. Two hours after infection, the MSCs were overlaid with a gel layer of methylcellulose, Matrigel and collagen (PureCol, Biomaterials, Fremont, USA) at a ratio of 1:1:1 in DMEM supplemented with 2% FCS. PaCaDD183 or MIA-PaCa2 spheroids were seeded on top of the gel layer and co-incubated with MSCs for 16 h. Next, the spheroids were transferred with a cut tip of a 10 μl pipette into new 96-well suspension culture plates. Forty-two hours after infection, images of non-fixed spheroids were acquired under a BIOREVO BZ-9000 microscope (Keyence, Neu-Isenburg, Germany).

### Immunohistochemistry and immunofluorescence staining

Paraffin-embedded 6-μm xenograft sections or spheroids fixed in 4% PFA and centrifuged in suspension onto slides in a CytoSpin 4 cytocentrifuge (Thermo Scientific, Schwerte, Germany) were stained as previously described [26]. Samples were stored at −20°C prior to further analysis. The primary antibodies were rabbit polyclonal antibodies against human c-Met (Enzo Life Science, Lörrach, Germany), cytokeratin 19, Ki67 (both from Abcam, Cambridge, UK), and the cleaved fragment of active human caspase-3 (R&D Systems, Abingdon, UK) and mouse monoclonal antibodies against human CD24 (SW11 hybridoma, kindly provided by Dr. P. Altevogt) and adenovirus-capsid (Merck Millipore, Darmstadt, Germany). Biotinylated goat anti-rabbit or anti-mouse IgG (Vector Laboratories, Peterborough, UK) was used as the secondary antibody for immunohistochemistry. The primary antibodies were omitted in the negative control. Goat anti-mouse Alexa Fluor 488 IgG and goat anti-rabbit Alexa Fluor 594 (Molecular Probes, Karlsruhe, Germany) were used as the secondary antibodies for immunofluorescence staining. The signals were detected with a Leica DMRB fluorescence microscope (Leica, Wetzlar; Germany). Images of representative fields were captured with a SPOT™ FLEX 15.2 64 Mp shifting pixel digital color camera (Diagnostic Instruments, Inc. USA) and analyzed with SPOT Basic/Advanced 4.6 software.

### Tumor xenotransplantation to the CAM of fertilized chick eggs

Tumor cells were transplanted to the chorioallantoic membrane (CAM) of fertilized eggs from genetically identical hybrid LB chicks, followed by treatment and humane euthanasia of the chick embryo as described [26, 48]. After resection, the tumor take rates were calculated from the percent of transplanted tumors that engrafted and the tumor volumes according to the formula Volume = 4/3 × π × r^3^ (r = 1/2 × square root of diameter 1 × diameter 2) [49]. Tumor tissue was fixed in Roti Histofix (Carl Roth, Karlsruhe, Germany) for 2-3 days and embedded in paraffin for further analysis.

### Statistical analysis

Experiments with established and primary PDA cell lines were performed three times with similar outcomes. *In vivo* transplantation experiments in fertilized chick eggs were performed twice with similar outcomes, and representative experiments are shown (15 to 20 eggs/group). The significance of the differences between the tumor volumes of OAd-infected tumors or tumors treated with adenovirus-infected MSCs were evaluated by Student's *t* test, or as shown in Figure 4, with a non-parametric Mann-Whitney test with Bonferroni-Holm correction for multiple testing. *P*<0.05 (*) and P<0.01 (**) were deemed statistically significant.

## SUPPLEMENTARY FIGURE



## Abbreviations

CSCs: Cancer stem cells
CAM: Chorioallantoic membrane
EMT: Epithelial mesenchymal transition
MSCs: Mesenchymal stromal cells
AOds: Oncolytic adenoviruses
PDA: Pancreatic ductal adenocarcinoma
