# Supplementary material for: Delivery of improved oncolytic adenoviruses by mesenchymal stromal cells for elimination of tumorigenic pancreatic cancer cells
Source: Oncotarget. 2016 Jan 27;7(8):9046–59. doi: 10.18632/oncotarget.7031 (PMC4891025; doi:10.18632/oncotarget.7031)
Supplement: Supplementary file 1 [file oncotarget-07-9046-s001.pdf]

SUPPLEMENTARY FIGURE

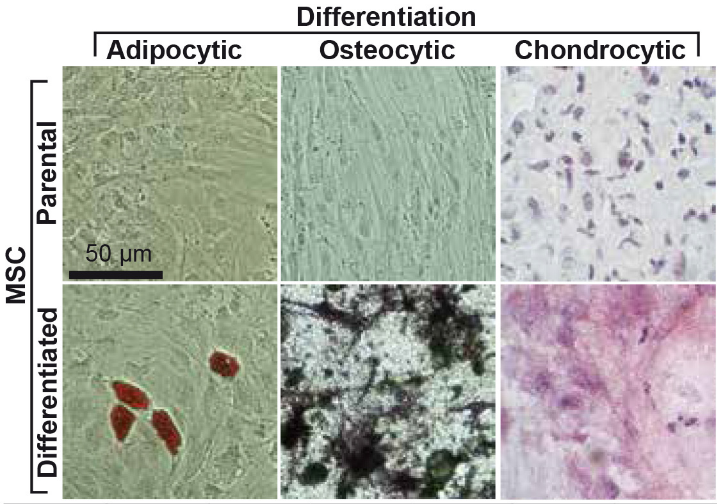

Marker	CD34	CD44	CD45	CD90	CD105	CD166
Exp	-	+	-	+	+	-

Supplementary Figure S1: Characterization of bone-marrow-derived MSCs. The functionality of human bone marrow-derived MSCs was evaluated by their differentiation potential. Parental MSCs were cultured in NH expansion medium (Parental) or in differentiation medium from kits for adipocytic, osteocytic or chondrocytic differentiation. Adipocytes, osteocytes, and chondrocytes were visualized by staining with OilRed O, BCIP/NBT, and Fast Green/Safranin O, respectively. To characterize the typical marker expression pattern, MSCs were stained with FITC-conjugated antibodies. FACS analysis indicated that the MSCs were positive for CD44, CD90 and CD105 (red) and negative for CD34, CD45 and CD166 (black), as expected.
